# The paracrine effect of exogenous growth hormone alleviates dysmorphogenesis caused by *tbx5* deficiency in zebrafish (*Danio rerio*) embryos

**DOI:** 10.1186/1423-0127-19-63

**Published:** 2012-07-09

**Authors:** Tzu-Chun Tsai, Jen-Kann Lu, Sie-Lin Choo, Shu-Yu Yeh, Ren-Bing Tang, Hsin-Yu Lee, Jen-Her Lu

**Affiliations:** 1 Department of Medical Research and Education, National Yang-Ming University Hospital, Yilan, Taiwan, Republic of China; 2 School of Medicine, National Yang Ming University, Taipei, Taiwan, Republic of China; 3 Laboratory of Molecular Biology, Institute of Aquaculture, National Taiwan Ocean University, Keelung, Taiwan, Republic of China; 4 Department of Pediatrics, Taipei Veterans General Hospital, Taipei, Taiwan, Republic of China; 5 Institute of Zoology, National Taiwan University, Taipei, Taiwan, Republic of China

**Keywords:** tbx5, Growth hormone, Apoptosis, Embryogenesis, Zebrafish

## Abstract

**Background:**

Dysmorphogenesis and multiple organ defects are well known in zebrafish (*Danio rerio*) embryos with T-box transcription factor 5 (*tbx5*) deficiencies, mimicking human Holt-Oram syndrome.

**Methods:**

Using an oligonucleotide-based microarray analysis to study the expression of special genes in *tbx5* morphants, we demonstrated that GH and some GH-related genes were markedly downregulated. Zebrafish embryos microinjected with *tbx5*-morpholino (MO) antisense RNA and mismatched antisense RNA in the 1-cell stage served as controls, while zebrafish embryos co-injected with exogenous growth hormone (GH) concomitant with *tbx5*-MO comprised the treatment group.

**Results:**

The attenuating effects of GH in *tbx5*-MO knockdown embryos were quantified and observed at 24, 30, 48, 72, and 96 h post-fertilization. Though the understanding of mechanisms involving GH in the *tbx5* functioning complex is limited, exogenous GH supplied to *tbx5* knockdown zebrafish embryos is able to enhance the expression of downstream mediators in the GH and insulin-like growth factor (IGF)-1 pathway, including *igf1*, *ghra*, and *ghrb*, and signal transductors (*erk1*, *akt2*), and eventually to correct dysmorphogenesis in various organs including the heart and pectoral fins. Supplementary GH also reduced apoptosis as determined by a TUNEL assay and decreased the expression of apoptosis-related genes and proteins (*bcl2* and *bad*) according to semiquantitative reverse-transcription polymerase chain reaction and immunohistochemical analysis, respectively, as well as improving cell cycle-related genes (*p27* and *cdk2*) and cardiomyogenetic genes (*amhc*, *vmhc*, and *cmlc2*).

**Conclusions:**

Based on our results, *tbx5* knockdown causes a pseudo GH deficiency in zebrafish during early embryonic stages, and supplementation of exogenous GH can partially restore dysmorphogenesis, apoptosis, cell growth inhibition, and abnormal cardiomyogenesis in *tbx5* knockdown zebrafish in a paracrine manner.

## Background

T-box transcription factor 5 (TBX5) is essential for cardiogenesis and forelimb development during embryogenesis in vertebrates. Mutation or haploinsufficiency of *tbx5* in humans is related to Holt-Oram syndrome (HOS), which features congenital heart defects and forelimb deformities [1,2]. The TBX5 protein was proven to be involved in determining early cell fate decisions, controlling differentiation and organogenesis, and regulating cardiac diastolic function in HOS patients [3,4]. In zebrafish, *tbx5* deficiency provokes cascading effects on multiple transcriptional expressions and causes extensive developmental retardation [5,6]. In developing zebrafish (*Danio rerio*) embryos, the *tbx5* gene is involved in the directed migration of individual lateral-plate mesodermal cells into future fin bud- and heart-producing regions [7], so embryos with the *tbx5* deficiency show anomalies in heart and pectoral fins that are identical to those in humans [6]. The *tbx5* deficiency also diminishes the expressions of *amhc*/*mhy6vmhc*, and *cmlc2*, causes heart defects, and is associated with pectoral fin anomalies and developmental delays [8,9]. Furthermore, TBX5 regulates organogenesis by modifying the activities of many transcription factors [8,10-12].

*Tbx5* also has essential roles in regulating progression of the cell cycle [13], cell growth, and apoptosis [14]. Blocking cell-cycle progression by *tbx5* depletion at the RNA level leads to a decrease in the cardiac cell number, an alteration in the timing of the cardiac differentiation program, defects in cardiac sarcomere formation, and ultimately cardiac programmed cell death [13,15].

GH belongs to the GH/prolactin (PRL) superfamily and functions by binding to homodimeric GH receptors. It is the major regulator of growth and is an important metabolic hormone [16]. Recent studies established that the GH/PRL superfamily is essential for organogenesis, such as that of the head, eyes, melanophores, and gas bladder in zebrafish [4]. Besides being implicated in growth, GH regulates gonad development, osmoregulation, and immunity in fish as well [17]. In GH-transgenic zebrafish, the overexpression of GH reduced the transcription of the antioxidant defense system and myogenesis-related genes [18], although the consequences of a shortage of GH in zebrafish embryos remain unknown.

Because the gain and loss of functions of GH in embryos cause developmental defects, GH is thought to play a vital role in embryogenesis. GH participates in embryonic development as a growth and differentiation factor, and in cell proliferation as an antiapoptotic factor and in meiotic progression [19,20]. Instead of pituitary GH, maternal or local GH takes part in regulating early embryogenesis *via* paracrine/autocrine effects, since GH and its receptors exist prior to the formation of functional pituitary somatotrophs [21,22].

Even though there is no evidence to date that shows any interaction between TBX5 and GH, both of them work with allied functions in regulating apoptosis, the cell cycle, and myogenesis during embryogenesis. Therefore, the role of GH during embryogenesis in embryos with congenital defects caused by an insufficiency of TBX5 remains undetermined but significant. In our study, GH was microinjected into zebrafish embryos at the 1 ~ 4-cell stages to reveal paracrine restoration effects from exogenous GH in *tbx5* morphants.

## Methods

### Animal ethics statement

Approval of this experiment was permitted by the Animal Ethics Review Board of National Taiwan Ocean University Aquaculture. Since zebrafish embryo under 7 day (168 hour post-fertilization) is excluded in the definition of "vertebrate animal" in review board, our study which used zebrafish embryo under 48 hpf was spared of regulation and review process of Basic Institutional Review Board (IRB).

### Maintenance of zebrafish

Zebrafish were maintained in 45-L aquaria heated to 28.5 °C with 25 fish per tank. The water was filtered, and about half of the water was replaced at least once a week. Adult zebrafish were fed 1 or 2 times per day with a variety of food, and the tank was cleaned by siphoning off any excess food after the second daily feeding. The day-night cycle was controlled with an automatic timer (14 h of light/10 h of dark).

### Breeding of zebrafish

Zebrafish reach sexual maturity in 10 ~ 12 weeks, but breeding fish should be 7 ~ 18 months of age for maximum embryo production. The day before breeding, 1/3 of the water was replaced and the tank was cleaned after feeding (1 ~ 2 h before the end of the light period). Finally, a collection box was placed at the bottom of the tank, and preparations were made to collect the embryos the next day.

### Embryo collection

We removed the collection box in the morning when the light was turned on and placed the collected embryos into an incubator maintained at a temperature of 28.5 °C.

### RNA isolation

Total RNA was isolated from 50 embryos using the guanidine isothiocyanate-based TRIzol solution. RNA samples were re-suspended in DEPC-treated water and quantified spectrophotometrically at 260 nm. The RNA quality was then checked by 1.2 % agarose gel electrophoresis, after staining with 1 μg/ml ethidium bromide. The RNA stock solution was stored at −80 °C.

### Microarray

Isolated total zebrafish embryo RNA was purified using an RNeasy® Mini Kit (QIAGEN, Hilden, Germany), and the quality was confirmed using an Aglient 2100 Bioanalyzer (Aglient Technologies, Santa Cruz, CA, USA). Purified RNA was reverse-transcribed into complementary (c)DNA using SuperScrip TM III RT (Invitrogen, Carlsbad, CA, USA). Before purifying and coupling the fluorescent dye using indirect cDNA labeling with a microarray kit (Invitrogen), the cDNA was hydrolyzed and neutralized using NaOH and HCl. The cDNA was then pretreated with GEx hybridization buffer (HI-PRM; Aglient Technologies) before transferring to hybridization chamber gasket slides for the hybridization reaction. The slide was scanned with an Axon Instruments GenePix 4000B scanner (Molecular Devices, Silicon Valley, CA, USA) and data analyzed with Genespring GX 10.0.2 (Aglient Technologies). All data is MIAME compliant and the raw data has been deposited in a GEO database (GSE33965) [NCBI tracking system #16217606].

### Semiquantitative reverse-transcriptase polymerase chain reaction (RT-PCR)

Total RNA was prepared from 50 defective or normal embryos (Invitrogen), with amplification of 3 μl of 1st-strand cDNA. Amplification primers for each specific mRNA deduced from published sequences were *igf1* (P1: 5’-TCTCATCCTCTTTCTCGC-3’, P2: 5’-GATAGTTTCTGCCCCC-3’), *ghra* (P1: 5’-AAGCATTGAGAGGTG-3’, P2: 5’-AGAGGAAGTGAGGAGAA-3’), *ghrb* (P1: 5’-GTTCCACCCGTTTTCA-3’, P2: 5’-GCGAGTCCTCATTCTGT-3’), *akt2* (P1: 5’-GAAGAGGATGAGCCAATG-3’ and P2: 5’-CTCCAACGCTGAAACAAT-3’), and *erk1* (P1: 5’-TCTGCCAATGTGCTGC-3’, P2: 5’-TGCCGTCTCCTCAAAG-3’). PCR conditions were comprised of denaturation at 95 °C for 3 min followed by 50 cycles of amplification (95 °C for 20 s, 59 °C for 15 s, and 72 °C for 20 s).

### Microinjection and morpholino (MO) treatment

The MO antisense oligonucleotide, *tbx5*-MO (5-GAAAGGTGTCTTCACTGTCCGCCAT-3), was designed against the *tbx5* translational start site (Gene Tools, Philomath, OR, USA). Wild-type (WT) embryos, primarily at the 1-cell stage with the chorion intact, were injected with 19.4 ng/4.3 nl of stock MO diluted in Danieau’s solution. Injected embryos were raised at 28.5 °C. Embryos used for analyzing the expression of various markers were fixed with 4 % paraformaldehyde. Otherwise, embryos were scored after 2 days of development for late effects. In our previous study, 4 control groups, including the 3' end of *tbx5*-MO(2) (5’-GCCTGTACGATGTCTACCGTGAGGC-3’), mismatched *tbx5*-MO (5’-GTCTCTTGACTCTCCGCGATCTCGG-3’), and embryos with blank microinjection and wild-types without microinjection, were included to identify the specific blockage of *tbx5* mRNA translation effect of *tbx5*-MO [9]. The efficacy and specificity of the *tbx5*-MO has been confirmed in previous published articles [9,14].

### Exogenous treatment with GH

Zebrafish embryos were micro-injected with 1 fM of human GH (Sigma-Aldrich, St. Louis, MO, USA) (*n* = 150/group, with triplicate determinations), accompanied by 19.4 ng/2.3 nl *tbx5*-MO at the 1-cell stage. Treated zebrafish embryos were placed into plates with wells, and their functional classification was evaluated at 30, 48, 72, and 96 h post-fertilization (hpf).

### Normal morphological growth rate assessment

Treated zebrafish embryos were placed into a plate with wells for longitudinal follow-up at 12 ~ 18-h intervals. The normal morphological growth rate was evaluated at 24, 30, and 48 hpf.

### Whole-mount *in situ* hybridization

Whole-mount *in situ* hybridization was performed as previously described by Schulte-Merker et al. [23]. The digoxigenin-labeled antisense full-length *amhcvmhc,* and *cmlc2* RNA probes were transcribed using T7 RNA polymerase (Promega, Madison, WI, USA). Whole-mount *in situ* hybridization was carried out essentially as described by Oxtoby and Jowett [24]. In brief, embryos were fixed with 4 % paraformaldehyde, digested with proteinase K, and hybridized with the zebrafish *amhcvmhc,* or *cmlc2* probes at 67 °C. An alkaline phosphatase-conjugated anti-digoxigenin antibody (Boehringer Mannheim, Dassel, Germany) was used to detect zebrafish *amhcvmhc,* or *cmlc2* signals. After staining with NBT/BCIP (Boehringer Mannheim), embryos were re-fixed with 4 % paraformaldehyde and stored in phosphate-buffered saline (PBS).

### Immunohistochemical analysis

Zebrafish embryos were fixed with 4 % paraformaldehyde in PBS. De-paraffinized sections (3 μm) of zebrafish embryo tissues were placed on slides and processed for immunohistochemistry. After application of a biotin blocking system (Dako, Glostrup, Denmark) for 30 min, sections were incubated with target-purified rabbit primary antibodies, including Bcl2, Bad, Cdk2, and P27 (all from Anaspec, Fremont, CA, USA) washed in PBS, and then incubated with a rhodamine-conjugated secondary antibody, goat anti-rabbit immunoglobulin G (IgG). After washing in PBS, sections were incubated with mounting medium and kept at 4 °C.

### Transmission electron microscopic (TEM) examinations

Embryos were fixed at 48 hpf with 2.5 % glutaraldehyde in Sorenson’s phosphate buffer, post-fixed with 1 % OsO_4_ in Sorenson’s phosphate buffer followed by dehydration through a graded series of ethanol washes, and embedded in Spurr’s EPON. Blocks were heated in an oven for 8 h at 70 °C. Semi-thin (1 μm) sections were cut and stained with toluidine blue for adequate preview under a microscope. Ultrathin sections (900 Å) were cut with a diamond knife, stained with uranyl acetate and lead citrate, and examined with an electron microscope.

### TdT-UTP nick end labeling (TUNEL) assay

Both whole mount and sectioned TUNEL assays were performed using an ApopTag kit (Chemicon, Heule, Belgium). Zebrafish embryos were briefly fixed with 4 % paraformaldehyde in PBS. Proteinase K-treated whole embryos or de-paraffinized sections (5 μm) of embryos were incubated with the TdT enzyme followed by anti-digoxigenin. Finally, embryos or slides were stained with DAB for 5 min.

### Western blot analysis

Embryos were homogenized on ice in lysis buffer (Sigma-Aldrich). Cellular debris was then pelletized by centrifugation at 12,000 rpm for 20 min, and the supernatant was collected and measured. Proteins were mixed with sample buffer before separation in 10 % sodium dodecylsulfate polyacrylamide electrophoresis (SDS-PAGE) gels. The SDS-PAGE was then transferred onto nitrocellulose membranes at 100 V for 1 h. Membranes were blocked with 5 % bovine serum albumin (BSA) buffer at room temperature for 1 h. The Akt and Erk primary antibody (Aviva Systems Biology, San Diego, CA, USA) was incubated overnight at 4 °C at a dilution of 1:1000. Nitrocellulose membranes were washed with PBST followed by incubation with a horseradish peroxidase (HRP)-conjugated secondary antibody (1:5000) for 1 h at room temperature before the images were digitized.

### Statistical analysis

Results are given as the mean ± S.D. Where applicable, Duncan’s new multiple range test was used to compare every pair of testing groups. Statistical significance was accepted at *p* < 0.05.

## Results

### The *tbx5* insufficiency causes morphological changes during zebrafish embryonic development

**Figure 1:**
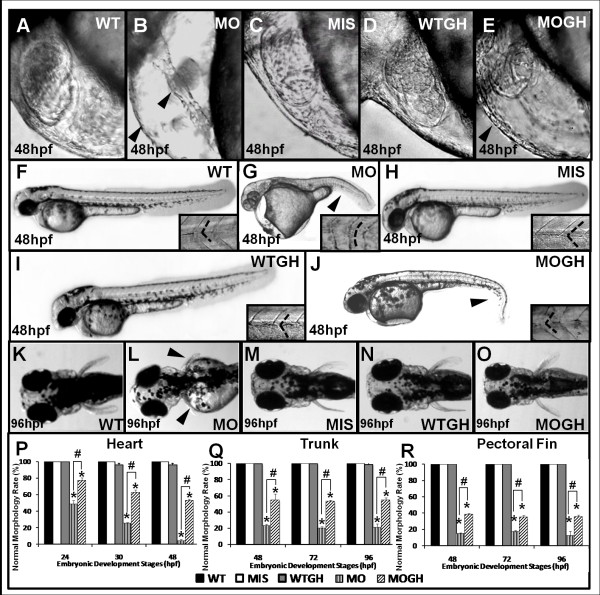
**Phenotypes of*tbx5*knockdown and GH-treated zebrafish embryos.** The normal appearance of hearts in wild-type (WT) (**A**) and MIS (**C**) group embryos and string-like hearts occurring in MO (**B**) group embryos are depicted. In WTGH embryos (**D**), hearts are identical to those of the WT (**A**) group, and hearts of MOGH group embryos (**E**) showed improvements. No significant differences were observed in trunks of WT (**F**), MIS (**H**), and WTGH group embryos (I), in which trunks were straight and somites appeared “V-shaped.” On the other hand, trunks of embryos injected with *tbx5*-MO were severely bent (**G**) and had “U-shaped” somites, but these were partially restored in MOGH group embryos (**J**). In the MO group (**L**), truncated or undeveloped pectoral fins were demonstrated; nevertheless, WT (**K**) embryos micro-injected with mismatched *tbx5*-MO (**M**), WT (**N**) exogenous GH–treated embryos, and *tbx5*-deficient embryos exhibited normal appearances. Statistically, the normal morphogenetic rates of the heart (**P**), trunk (**Q**), and pectoral fins (**R**) were significantly lower in the MO group and partially improved in the MOGH group. Defective embryos were not found in the WT or MIS groups and almost all of the embryos in the WTGH group developed properly. Data are presented as mean ± S.D. *p < 0.05 *vs.* WT; ^#^*p* < 0.05 MO + GH *vs.* MO. Black arrowhead, defect site; dashed line, shape of somite border; MO, *tbx5* knockdown; MIS, mismatch *tbx5*-MO-treated embryos; WTGH, WT embryos treated with GH; MOGH, *tbx5*-MO- and GH-treated embryos.

At 48 hpf, looped hearts with apparent chambers (atria and ventricle) were observed in WT zebrafish embryos (Figure 1A), their trunks appeared straight without bending, and somites were “V-shaped” (Figure 1F). At 96 hpf, they displayed well-formed pairs of pectoral fins (Figure. 1K). Perturbations of cardiac development (Figure 1B) were exhibited in *tbx5*-MO treated (MO) zebrafish embryos, along with curved trunks and abnormal “U-shaped” somites (Figure 1G), and pectoral fin growth (Figure 1L) was either truncated or even atretic. Moreover, the defect rates in the heart (Figure 1P), trunk (Fig. 1Q), and pectoral fins (Figure 1R) were very high in the MO group compared to those of the WT and mismatch *tbx5*-MO-treated (MIS) groups. Formation of the heart (Figure 1C), trunk (Figure 1H), and pectoral fins (Figure 1M) in the MIS and WT groups was similar.

### Multiple growth-related genes were downregulated in *tbx5* knockdown embryos

We screened growth-related genes that were downregulated after knockdown of *tbx5* using a zebrafish microarray. Genes that were 1.5× downregulated were included. Multiple genes participating in growth were downregulated in zebrafish embryos with the *tbx5* deficiency (Table 1). Growth-related genes *igfbp2b*, *ghra*, *ing4*, *mdkb*, *grb2*, *vegfaa,* and *fibp1* were downregulated at the heart-tube stage at 24 hpf. At 30 hpf, when the heart begins to loop, *pdgfab*, *gh1*, *fgf1*, *fgf6a,* and *vegfab* were downregulated in *tbx5* knockdown embryos. Furthermore, *gata5* and *ghr1* were found to be downregulated at 48 hpf.

**Table 1:** **1.5× down regulated growth -related genes in*tbx5*knockdown embryos in different embryonic developmental stages**

**Gene Symbol**	**Gene Name**	**Genbank #**	**Function**	**Stage (hpf)**	**Reference**
*igfbp2b*	insulin-like growth factor protein 2b	NM_131458	general embryonic development and growth, regulating vascular development	24	Zhou, 2008
*ghra*	growth hormone receptor a	NM_001083578	cytokine receptor activity	24	Di Prinzio, 2010
*ing4*	inhibitor of growth family, member 4	NM_001020468	regulating brain tumour growth and angiogenesis	24	Susan Nozell, 2008
*mdkb*	midkine-related growth factor b	NM_131716	brain development, neural crest formation	24	Liedtke, 2008
*grb2*	growth factor receptor-bound protein 2	NM_213035	distinct effects on neural crest and floorplate development	24	Ryan P Million, 2004
*vegfaa*	vascular endothelial growth factor Aa	AF016244	blood vessel endothelial cell proliferation	24	Bahary, 2007
*fibpl*	fibroblast growth factor (acidic) intracellular binding protein, like	NM_212861	Kupffer's vesicle development	24	Hong, 2009
*pdgfab*	platelet derived growth factor alpha b	NM_001076757	positive regulation of cell division	30	Eberhart, 2008
*gh1*	growth hormone 1	NM_001020492	growth control	30	Toro, 2009
*fgf1*	fibroblast growth factor 1	NM_200760	hemopoiesis	30	Songhet, 2007
*vegfab*	vascular endothelial growth factor Ab	NM_001044855	angiogenesis	30	Bahary, 2007
*gata5*	GATA-binding protein 5	NM_131235	specification of cardiomyocytes	48	Holtzinger, 2007
*ghrl*	ghrelin/obestatin preprohormone	NM_001083872.1	encodes ghrelin-obestatin preproprotein	48	Li, 2009

### Exogenous GH-improved embryonic defects in zebrafish with *tbx5* deficiency

Data from microarray screening showed that GH was downregulated in the early embryonic stages in zebrafish embryos with the *tbx5* deficiency. WT embryos did not show defects of the heart (Figure 1P), pectoral fins (Figure 1Q), or trunk (Figure 1R). Microinjection of GH into *tbx5*-MO treated embryos (MOGH group) caused defects of the heart (22.7 % at 24 hpf, 37.3 % at 30 hpf, and 46.7 % at 48 hpf; Figure 1P), pectoral fins (61.3 % at 24 hpf, 64.7 % at 30 hpf, and 65.3 % at 48 hpf; Figure 1Q), and trunk (45.3 % at 24 hpf, 45.3 % at 30 hpf, and 46.7 % at 48 hpf; Figure 1R).

Microinjection of *tbx5*-MO into WT embryos (the MO group) caused specific defects of the heart (string-like heart, cardiac edema, and loss of ventricular contractility) (51.3 % at 24 hpf, 74.7 % at 30 hpf, and 96 % at 48 hpf; Figure 1P), pectoral fins (85.3 % at 24 hpf, 88 % at 30 hpf, and 88 % at 48 hpf; Figure 1Q), and trunk (76 % at 24 hpf, 80 % at 30 hpf, and 78.7 % at 48 hpf; Figure 1R). Comparing the MO group to the MOGH group, the incidence of embryonic defects due to *tbx5* insufficiency was significantly reduced (Figure 1P-R).

Compared to the WT group, microinjection of exogeneous GH into WT embryos (WTGH) did not cause significant phenotypic changes, and no embryonic defects were identified (Figure 1P-R). There were no statistical differences in embryonic defects between the WT group (*n* = 50) and either the MIS group (*n* = 50) or the WTGH group (*n* = 50).

### The paracrinous effect of exogenous GH in activating the IGF-1 pathway

**Figure 2:**
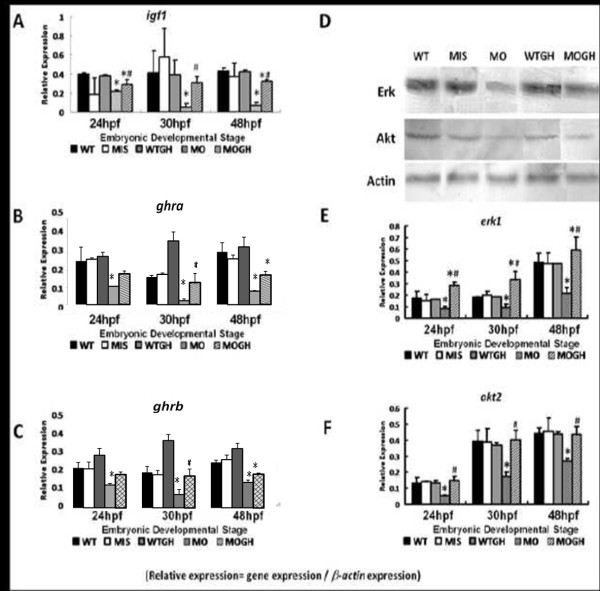
**Expressions of*igf1*,*ghra*,*ghrb*,*akt2,*and*erk1*in*tbx5*knockdown embryos.** (**A**) The expression of *igf1* was significantly reduced in MO group embryos throughout the developmental stages. Its expression in WTGH and MIS embryos was similar to the control and wild-type (WT) groups. The expression of *igf1* was significantly restored in the WTGH treatment group. Expressions of GH receptors *ghra* (**B**) and *ghrb* (**C**) were significantly depressed in MO embryos but restored in the MOGH treatment group. (**D**) In the Western blot analysis, protein expressions of Akt and Erk in MO zebrafish embryos were depressed, but expressions were similar among the WT, MIS, and WTGH groups at 30 h post-fertilization (hpf). Expressions of Akt and Erk were restored in the MOGH group. In the semiquantitative PCR analysis, mRNA expressions of *erk1* (**E**) and *akt2* (**F**) were significantly reduced in MO group embryos and restored in the MOGH group. Data are presented as the mean ± S.D. *p < 0.05 *vs.* WT; ^#^*p* < 0.05 MOGH *vs.* MO. MO, *tbx5* knockdown; MIS, mismatched *tbx5*-MO-treated embryos; WTGH, WT embryos treated with growth hormone (GH); MOGH, *tbx5*-MO- and GH-treated embryos.

Genes participating in the GH/IGF-1 pathways, *igf1* (Figure 2A), *ghra* (Figure 2B), and *ghrb* (Figure 2C), were downregulated in the MO group but were partly reactivated in the study groups simultaneously treated with GH (Figure 2A-C). On the other hand, genes participating in the GH/IGF-1 pathway showed no statistically significant differences in the WT group and MIS group (Figure 2A, B, C).

Since the affected receptors function through phosphorylation, we examined the expression of phosphorylation-related genes, *erk1* and *akt2,* at the protein and gene levels using Western blot and semiquantitative RT-PCR, respectively. Gene expression levels of *erk1* (Figure 2E) and *akt2* (Figure 2F) could be identified at 24, 30, and 48 hpf, but were depressed in embryos with the *tbx5* deficiency. Exogenous GH increased the expression of genes and proteins in embryos with the *tbx5* deficiency, but these expressions caused no significant changes in the MIS group (Figure 2E, F). GH downstream factors Erk and Akt, however, were significantly reduced in the MO group, and their gene expressions were improved in the MOGH group.

Expressions of the phosphorylation-related genes of *erk1* and *akt2* in the WT, MIS, and WTGH microinjection groups were similar (Figure 2D-F).

### Exogenous GH partially normalized the apoptotic effect induced by *tbx5* deficiency

**Figure 3:**
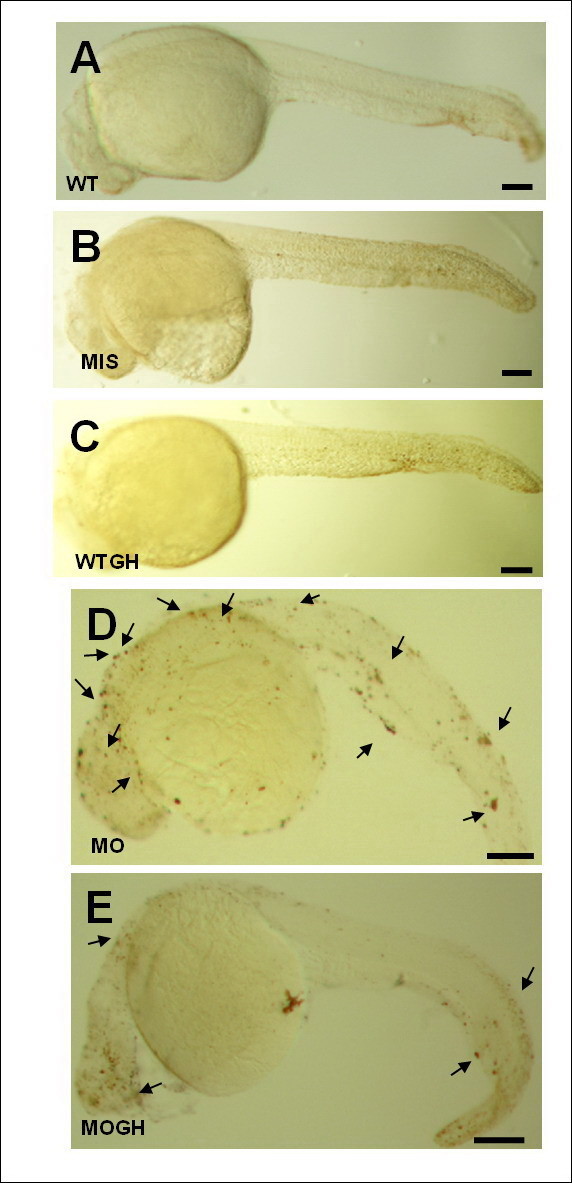
**Growth hormone (GH)-treated*tbx5*-knockdown zebrafish embryos show reduced apoptosis at 30 h post-fertilization (hpf).** A TUNEL assay revealed no apoptotic spots in WT (**A**), MIS (**B**), and WTGH (**C**) embryos. (**D**) However, massive apoptotic spots were visible in MO embryos. (**E**) In the MOGH group, apoptotic sites were reduced. (**A**-**E**) Embryo anteriors are to the left. Scale bar = 0.1 cm. Black arrow, apoptotic site; WT, wild-type embryos; MO, *tbx5* knockdown; MIS, mismatched *tbx5*-MO-treated embryos; WTGH, WT embryos treated with GH; MOGH, *tbx5*-MO- and GH-treated embryos.

The TUNEL assay demonstrated only few apoptotic spots in WT (Figure 3A), MIS (Figure 3B), and WTGH embryos (Figure 3C). Apoptotic spots were significantly induced in the MO group (Figure 3D) and were diminished in the MOGH-treated group (Figure 3E).

**Figure 4:**
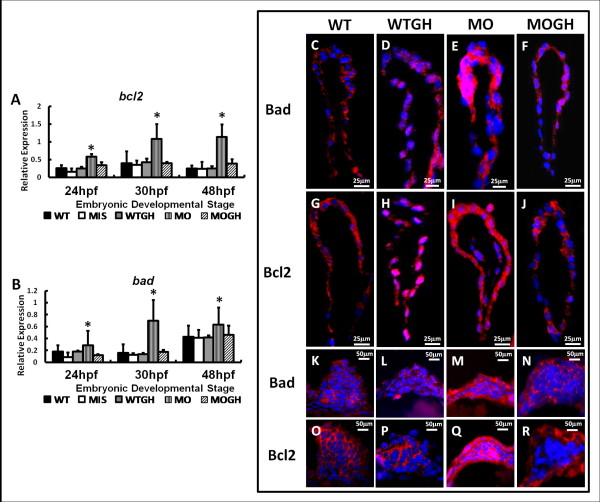
**Expression of apoptosis-related genes and proteins in*tbx5*knockdown and growth hormone (GH)-treated zebrafish embryos at 30 h post-fertilization (hpf).** Using a semiquantitative RT-PCR, the apoptotic genes, *bcl2* (**A**) and *bad* (**B**), were significantly induced in MO group embryos and showed no significant differences among the WT, WTGH, and MOGH groups. (*n* = 3, 50 embryos/stage; relative expression = gene expression/*β*-*actin* expression). (**C**-**R**) Zebrafish embryos were stained by apoptosis-related antibodies, Bad and Bcl2 (red), and counterstained with DAPI (blue) for nucleus observation. In sagittal sections of the heart, Bad and Bcl2 were similarly expressed and significantly induced in *tbx5-*deficienct embryos (**E**, **I**), and showed no significant differences among the WT (**C**, **G**), WTGH (**D**, **H**), and MOGH (F, J) groups. Transverse sections showed that expression patterns of Bad and Bcl2 in pectoral fins were significantly induced in *tbx5*-deficient embryos (**M**, **Q**) and showed insignificant differences among the WT (**K**, **O**), WTGH (**L**, **P**), and MOGH (N, R) groups. (**C**-**R**) Embryo anteriors are to the left. WT, wild-type embryos; MO, *tbx5* knockdown; MIS, mismatch *tbx5*-MO-treated embryos; WTGH, WT embryos treated with GH; MOGH, *tbx5*-MO- and GH-treated embryos. Data are presented as the mean ± S.D. *p < 0.05 *vs.* WT

**Figure 5:**
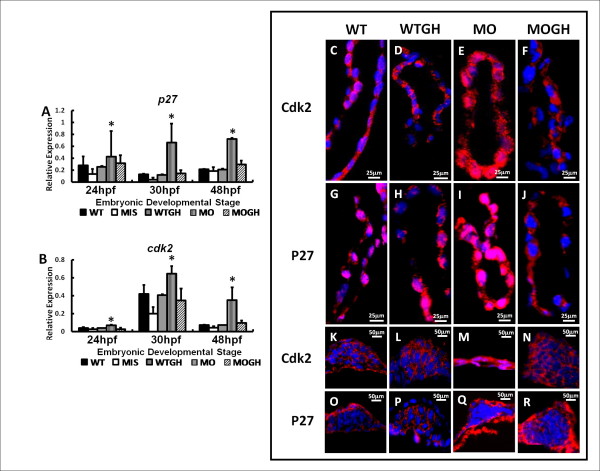
**Expression of cell cycle-related genes and proteins in*tbx5*knockdown and GH-treated zebrafish embryos at 30 h post-fertilization (hpf).** Cell cycle-related genes, *p27* (**A**) and *cdk2* (**B**), were significantly induced in MO group embryos throughout the developmental stages but were similar among the WT, WTGH, and MOGH groups. (*n* = 3, 50 embryos/stage; relative expression = gene expression/β-actin expression). (**C**-**R**) Zebrafish embryos was stained with cell cycle-related (Cdk2 and P27) antibodies (red) and counterstained with DAPI (blue) for nuclear observation. In sagittal sections of the heart and transverse sections of the pectoral fins, expression patterns of Cdk2 and P27 were similar in all treatment groups in that they were significantly induced in *tbx5*-deficienct embryos (**E**, **I**, **M**, **Q**) and showed insignificant differences among the WT (**C**, **G**, **K**, **O**), WTGH (**D**, **H**, **L**, **P**), and MOGH (**F**, **J**, **N**, **R**) groups. (**C**-**R**) The anteriors of the embryos are to the left. WT, wild-type embryos; MO, *tbx5* knockdown; MIS, mismatch *tbx5*-MO-treated embryos; WTGH, WT embryos treated with growth hormone (GH); MOGH, *tbx5*-MO- and GH-treated embryos. Data are presented as the mean ± S.D. *p < 0.05.

We analyzed the expressions of cell apoptosis-related genes at the RNA and protein levels. Our results showed a remarkable increase of *bcl2* (Figure 4A) and *bad* (Figure 4B) in MO group embryos in all studied periods. However, compared to gene expression in the WT group, no significant changes were found in embryos injected with either MIS or WTGH (Figure 5A, B). Overexpression of the *bad* and *bcl2* genes was then confirmed by analyzing their protein expressions by performing immunohistochemical analyses in the heart and pectoral fins at 30 hpf. Bad and Bcl2 genes showed identical protein expression patterns, which were observed in messenger (m)RNA expression analysis in the heart and pectoral fins (Figure 4C-R). Mild expressions of the apoptosis-related proteins, Bad and Bcl2, were observed in the heart (Figure 4C, G) and pectoral fins (Figure 4K, 4O) of WT embryos, and increased expressions of Bad and Bcl2 were detected in the heart (Figure 4E, I) and pectoral fins (Figure 4M, Q) of *tbx5* knockdown embryos. Expression levels of apoptosis-related proteins were observed in the heart (Figure 4D, F, H, J) and pectoral fins (Figure. 4L, N, P, R) of the WTGH and MOGH groups.

### Effect of exogenous GH on genes related to the cell cycle

Depletion of *tbx5* caused an increase in the expressions of S stage-related mRNA *p27* and *cdk2* (Figure 5A, B) in *tbx5* morphants. However, in the MOGH group, expressions of *p27* and *cdk2* were partially restored, which showed no significant difference compared to the WT or MIS groups (Figure 5A, B).

mRNA expressions of cell cycle-related genes were confirmed by performing an immunohistochemical analysis. Protein expressions of Cdk2 and P27 in the heart and pectoral fins had similar patterns as observed in the mRNA expression analysis (Figure 5C-R). Expressions of cell cycle-related proteins, Cdk2 and P27, were observed in the heart (Figure. 5C, G) and pectoral fins (Figure 5K, O) of WT embryos. Expressions of Cdk2 and P27 were induced in the heart (Figure. 5E, I) and pectoral fins (Figure 5M, Q) of MO-group embryos. Protein expression levels of Cdk2 and P27 were identical in the heart (Figure 5D, 5 F, 5 H, 5 J) and pectoral fins (Figure 5L, N, P, R) of the WTGH and MOGH groups.

### Exogenous GH improves the expression of cardiomyogenesis genes in *tbx5* knockdown embryos

**Figure 6:**
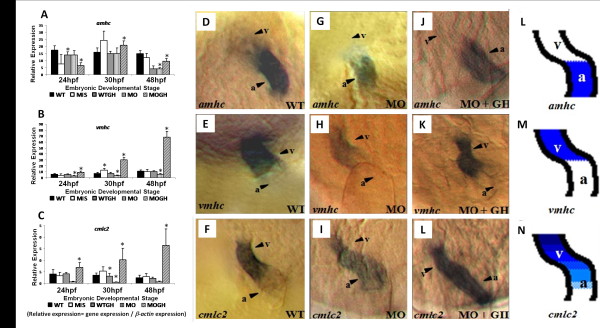
**Expressions of cardiomyogenesis-related genes in GH-treated*tbx5*mutants. Expressions of (A)*amhc*, (B)*vmhc,*and (C)*cmlc2*in MO group zebrafish embryos were depressed throughout the developmental stages compared to WT zebrafish embryos.** Expression of these thre cardiac myogenesis genes showed significant restorative effects in MOGH group embryos. On the other hand, expressions of cardiac myogenesis genes were not significantly affected in WTGH group embryos. The expression of *amhc* was restricted to the atrium in WT embryos (**D**), and its expression was depressed in MO group embryos (**G**). (**J**) MOGH group zebrafish embryos showed an ameliorating effect on restoration by the expression of *amhc*. The expression of *vmhc* was restricted to the ventricle in WT embryos (**E**) but showed minor expression in MO group embryos (**H**). (**K**) However, the expression of *vmhc* was significantly induced in the MOGH group. The expression pattern of *cmlc2* was similar to that of *vmhc,* in that expression was low in *tbx5*-deficient embryos (I) but was restored in GH-treated embryos (**L**). Schematics indicate the location of the expressions of *amhc* (**L**), *vmhc* (**M**), and *cmlc2* (**N**). Data are presented as the mean ± S.D. *p < 0.05 *vs.* WT. WT, wild-type embryos; MO, *tbx5* knockdown; MIS, mismatched *tbx5*-MO-treated embryos; WTGH, WT embryos treated with growth hormone (GH); MOGH, *tbx5*-MO- and GH-treated embryos; a, atrium; v, ventricle.

The result of whole-mount *in situ* hybridization demonstrated that the expressions of *amhc*, *vmhc,* and *cmlc2* were reduced in *tbx5* knockdown embryos (Figure 6G-I) compared to WT embryos (Figure 6D-F); however, in the MOGH group, expressions of *amhc* (Figure. 6J), *vmhc* (Figure 6K), and *cmlc2* (Figure 6L) were improved. In a semiquantitative RT-PCR test, the expression of *amhc* was repressed at 48 hpf in the MO group and was improved in the MOGH treatment group (Figure 6A). On the other hand, expressions of *vmhc* (Figure 6B) and *cmlc2* (Figure 6C) were significantly repressed in all developmental stages in *tbx5* knockdown embryos but were significantly improved in the MOGH-treated group. Their expressions in the MIS, WTGH, and WT groups were similar (Figure 6A-C).

## Discussion

Significant dysmorphogenesis (Figure 1) shown in the MO group is similar to congenital defects in humans with HOS. The defects of a string heart [6,10,11,15,25-29] and dysgenetic fins [30-32] are well studied in several species models with *tbx5* insufficiency, especially zebrafish. We also explored those genes associated with altered *tbx5* levels during embryogenesis and those which contribute to developmental defects. We investigated gene expressions in dysmorphogenesis of a zebrafish *tbx5-*deficient model in order to reveal the role of *tbx5* in altering transcription using an oligonucleotide-based microarray analysis, as it is sensitive to a single injection of *tbx5* morpholino in zebrafish embryos. Our microarray analysis results identified many genes with different functions and different categories that were up- or downregulated in zebrafish embryos with *tbx5* deficiency by morpholino in the early stages of organogenesis. Among these genes, some associated with growth, including *igfpb2bgh1ghr1,* and *ghra*, which occur respectively during different time windows of embryogenesis and are normally activated during different stages of early organogenesis, were appreciably depressed in embryos with *tbx5* deficiency [32-35]. A deficiency in *tbx5* leads to multiple organ defects including the heart, trunk, and pectoral fins, and also decreases the expressions of *gh1* (*gh*), and *ghra*[32,36]. Our data also revealed that knockdown of *tbx5* in embryos diminished GH/IGF-1 pathway mediators, including GH, IGF-1, and GH receptors.

Exogenous GH was used in our study to partially restore the anomalies during embryogenesis to ensure the involvement of GH in multiple organ defects by tbx5 knockdown. In the study, we injected human recombinant GH, which human GH instead of zebrafish GH is reported to have full activity in fish as well as in human [37], into the yolk of the zebrafish embryos as an alternative of soaking the embryos with GH-contain water. Partially because the GH containing water solution is difficult to control the concentration, partially microinjection remains the most effective methods to introduce DNA, RNA, and proteins into fertilized zebrafish eggs and embryos [38]. Theoretically, the recombinant GH could be successfully delivered into the yolk of 1–2 cell stages and diffused into most embryonic cells of blastomere *via* intercellular substance. The expression of *igf1ghraghrberk1*, and *akt2* genes was increasing after co-injection with GH and tbx5 morpholino at 1-cell stage. These results revealed the exogenous GH has activated the downstream signaling pathway. However, it is still unknown how and where the exogenous GH binds with the GH receptors. Whether the injected exogenous human GH is packaged and sent outward to bind with the membranous GH receptors, or it directly binds with the cytoplasmic GH receptors, remains unidentified because the expression of GH receptors could be either in the nucleus or cytoplasm, or both, in different embryonic tissues and cells [39].

Exogenous GH in zebrafish embryos with *tbx5* deficiency could activate expression of GH receptor genes, to induce an increase in *igf1* levels, and to elevate downstream Akt and Erk systems, coinciding with restoration of morphological anomalies and transcriptional cascades. It could be hypothesized that GH is a factor that may act in a paracrine manner within the *tbx5* functional pathway to modulate embryonic development.

*Tbx5* is essential for regulating the progression of the cell cycle by controlling the length of the embryonic cardiac cell cycle [13] and regulating apoptosis in endocardial cells, myocardial cells and the *septum primum*[40], all of which contribute to abnormal cardiogenesis. GH influences the growth of embryonic cells and modulates embryo cell cycle and proapoptotic metabolism [41]. In our study, exogenous GH partially restored the expression of *tbx5* downregulated genes, which contributes to developmental delays in organogenesis including the cell cycle (*p27* and *cdk2*) and apoptosis (*bcl2* and *bad*). Early administration of exogenous GH improves the outcome of *tbx5*-deficiency-mediated heart defect embryos probably by inducing cardiac cells to re-enter the cell cycle. It also reduces aberrant apoptosis because GH works in a similar way to stimulate the cardiomyocyte to re-enter the cell cycle and thereby increases the number of cardiac myocytes in ischemic and infarcted myocardia [3,26].

The GH signaling pathway governs cell growth, proliferation, and apoptosis by controlling key regulatory genes that execute these processes. Herein, we also provide the first evidence that tbx5 acts together with GH to regulate cardiac myogenetic pathway-responsive genes (*cmlc2*, *amhc*, and *vmhc*). Exogenous GH restored the expression levels of *amhc*, *vmhc*, and *cmlc2* in our *tbx5* morphant embryos with cardiac defects. Inactivation of *tbx5* diminished *amhc*, *vmhc*, and *cmlc2* expressions, and although it also reduced heart size, exogenous GH reversed that result and enhanced cardiac formation in zebrafish embryos. Our results indicate not only that GH is necessary for the growth of these cardiac structures, but also that supplementary exogenous GH restores tbx5 knockdown-mediated defects, including dysmorphogenesis and cardiomyogenesis, *via* cell proliferative and apoptotic pathways. It could be concluded that knockdown of tbx5 in early zebrafish embryogenesis causes functional GH deficiency and leads to dysmorphogenesis. The comorbidity of morphologic defects and functional GH deficiency could be observed in early embryogenesis of *tbx5* morphants and implied that GH may involve a role in embryogenesis including cardiomyogenesis through transcriptional regulation of *tbx5*.

We found no literature underlining the interaction or the relationship between *tbx5* and growth-related genes shown in Table 1. This is especially true for GH. TBX5 is a member of the T-box transcription factor family. It has a sequence-specific DNA-binding site that improves an inducible recognition element of TBX5 that binds to a specific DNA sequence [42]. Thus, *tbx5* synergistically activates transcriptional regulation of downstream gene expression and controls the transcription of genetic information in embryonic development. In many *tbx5* mutants, affinities bound to a specific DNA target site were reduced by a variable amount, and even the ability to bind nonspecific DNA differs. Both contribute to the misregulation of target gene expression.

GH exerts different actions in different tissues through a complex functioning pathway involving many growth factors and their receptors [43]. It is mainly supposed to act through mediation of the GH/IGF-1 pathway, including GH receptors and cytoplasmic and intranuclear factors. Though the relationship between tbx5 and GH remains undetermined, there exist some interactions or association between them during zebrafish embryogenesis. According to the recent studies, we could reasonably assume that transcription factors, probably including TBX5, may play a role in interacting with the GH mediator array in the nucleus. For example, GH-responsive transcription factors in sex-specific liver gene expressions are an example of interaction between GH and transcription factors in specific tissues [44]. Meanwhile, a transcription factor that regulates GH-variant gene expression could also exist [45]. On the other hand, the latest investigation declared, the transcription factor STAT3 (signal transducer and activator of transcription 3), one of downstream signaling molecules for GH, directly control the expression of tbx5 in P19CL6 cells for cardiomyocyte differentiation [46]. The conclusion not only points out a connection between GH and TBX5 transcription factor, but also implies that GH might activate and increase *tbx5* expression in *tbx5* morphants. That is, GH compensates the deficiency of TBX5 *via* STAT3 and other transcriptional factors and maintains the *tbx5*-associated cascade effects of organogenesis and morphogenesis in early embryonic stages, partially.

Though GH is generally considered to be an endocrine factor because it is primarily synthesized by pituitary somatotrophs and is secreted into the circulation. However, it has been clear that GH is produced in many tissues outside the pituitary gland and acts as a local or maternal growth factor in the autocrine/paracrine regulation of cellular differentiation during embryonic and fetal development [22,47]. Because the growth effect occurs prior to differentiation of pituitary somatotrophs, early embryonic growth is independent of pituitary GH. Prinzio *et al.* announces the distribution and expression of growth hormone receptors, *ghra* and *ghrb*, in embryonic zebrafish by means of RT-PCR and whole mount *in situ* hybridization, and the genomic organization by cloning and sequence analysis [32]. They prove *ghra* and *ghrb* expression was detected at all stages entail maternal origin [32]. It is undeniable that maternal GH did play a role in early embryonic development of zebrafish; and it is taken for granted that blockage of GH directly interferes with normal cardiac development and even induces cardiac malformation and dysfunction [48]. If we hypothesized that Tbx5 may play a role in interaction with maternal/local GH and in the activation of cascade GH signaling in early embryonic development to assist the chronological organogenesis. Then it is reasonable that the normal physical responses to maternal GH in embryonic zebrafish development is decreased after knockdown of tbx5, and the responses could be reinforced by extra supplement of passable exogenous GH.

Phosphorylation that is mediated by PI3K-AKT and MAPK signaling cascades is an important component of the acting mechanism of local GH-stimulated transcription at the organogenesis phase [49-51]. Our results suggest that the local GH pathway acts similarly to the conventional GH/IGF-1 signaling pathway [48,52-54] and that exogenous GH activates Akt and Erk pathways in the nucleus, probably by binding to local insulin receptors. Local GH signaling downstream of the P13K-AKT system is a key effect related to regulation of cell survival and mRNA translation, while signaling downstream of the MAPK-ERK system involves regulating cellular proliferation. This suggests that exogenous GH signaling occurs *via* local GH receptors during heart looping formation and chamber maturation stages.

We established four control groups in order to verify the specific GH effects without interference of morpholino and technical influences of micro-injection. However, interesting phenomena were disclosed by careful interpretation among those different control groups. First, *igf1* transiently surged in zebrafish embryos injected by missense morpholino (MISMO group) without subsequent effects, but the phenomenon didn’t happen in the expressions of *ghra*, *ghrb*, *erk1*, and *akt2*. It may aggressively assume that GH effects may involve IGF-1 dependent and independent pathways in embryonic development to switch on the cascade reactions. Thus the role of IGF-1 attracts attention for further exploration. Moreover, the expressions of *ghra* and *ghrb* in WTGH group significantly increased than MOGH group, but the expressions of *erk1*, *akt2*, *amhc*, *vmhc*, *cmlc2* in WTGH group had no remarkable change than MOGH group. It implies that excess GH works inefficiently in individuals without tbx5 deficiency, or GH deficiency. GH receptors could be reactivated and responded to exogenous GH, but downstream signals and cardiomyogenesis-related genes didn’t markedly act in response to overload of GH. The results are compatible with the biological functions of GH in mature adult individuals. Surly, supplementary designs of control groups, for example, such as use of BSA with equivalent amount as a blank control, could be launched to access the authentic GH effects by our experimental model.

Although GH is able to partially restore dysmorphogenesis and cascade gene expressions in *tbx5* morphants, it cannot completely rescue those changes. Proper timing of GH treatment and optimal dosing might be found to enhance its therapeutic capability. Conversely, this approach may be limited by GH being partially significant to the complex functioning of the *tbx5* network or because it compensates for only a small part of the chronological effects of *tbx5* deficiency. Additional research is required to determine whether it is practicable to introduce GH to mend developmental defects in early embryogenesis.

## Conclusions

In summary, our work provides novel insights into the possible role of GH in contributing to developmental defects in zebrafish embryos with *tbx5* deficiency. We suggest that the functional knockdown of zebrafish *tbx5* results in a failure to develop a complete or functional heart, trunk, and pectoral fins and might be due to a functional GH deficiency induced by the *tbx5* deficiency because it is a key factor causing abnormal organogenesis. Exogenous GH experiments in zebrafish embryos with *tbx5* deficiency led to the conclusion that intrinsic growth-control mechanisms, including apoptosis, cell cycle, and cardiomyogenesis that control organic growth, depend on local GH and growth factors between cells and their neighbors. They also indicate that these interactions include controlling cardiac loop formation and the development of trunk and pectoral fins. The improvement of abnormal embryonic organogenesis in zebrafish embryos with *tbx5* deficiency by the administration of exogenous GH suggests its potential application in human congenital anomalies.

## Competing interests

The authors declare that they have no competing interests.

## Authors’ contributions

JHL and JKL conceived of the study, participated in its design, coordination. TCT participated in its design and drafted the manuscript. SLC and SYY carried out the molecular genetic studies. RBT and HYL participated in its design and coordination. All authors read and approved the final manuscript.
